# Original Vision of the JACMP

**DOI:** 10.1120/jacmp.v14i1.4246

**Published:** 2012-01-07

Greetings to the *JACMP* Community! In search of inspiration for my inaugural editorial of my second round as Editor‐in‐Chief of the *JACMP*, I looked at some historical documents for guidance. The idea for an open‐access journal (that term did not exist at the time) was first discussed as I remember in November of 1997. Ann Wright had mentioned to me, in an almost offhanded way, that it would be very useful to have a standalone clinical medical physics journal, apart from *Medical Physics*. She was thinking of a print journal, but it occurred to me that the Internet (then only a few years old) would offer a great opportunity to publish a new type of journal — one that did not offer print issues, but would exist only online. The savings of printing and postage would allow for a new type of business model, one that could provide the articles at low or even at no cost to the author and reader.

Alex Turner, ACMP Chairman, thought that was an idea that should be explored and appointed a task group to investigate the possibility of a new *Journal for Clinical Physics*. We had no concept of how difficult and complex it would be to realize this vision, or of the dearth of resources available to proceed. The following is the verbatim initial report of that task group as written in April of 1998. It shows, I believe, that we formulated the kernel of a vision that was later substantially realized, although we did get more than a few things wrong.

In 2000, the *JACMP* became, I believe, the first open‐source and open‐access academic journal in Radiology and Radiation Oncology, and one of the first such journals in Medicine. Next issue, I will discuss how our vision for the *JACMP* has evolved over the years, and what we might expect for the future.

Michael D. Mills, Ph.D

Editor‐in‐Chief

October 8, 2012

## TAsk GrOuP fOr A JOurnAl Of CliniCAl PhysiCs

**Table:** 

Report: 4/17/98			
Michael Mills, Chair	Bruce Curran	Jeff Fairbanks	John Glover
Kenneth Hogstrom	Colin Orton	John Pacyniak	Alex Turner

## I. INTRODUCTION

The idea that the medical physics community would be served by a clinical medical physics journal is not new. For some time, medical physicists involved primarily in clinical work have found it difficult to identify a forum which would allow for timely, wide, and archival distribution of clinical techniques and information. The advent of Web‐based technologies and the growing access of the medical physics community to the Web‐based information pool suggest an electronic journal is an idea worthy of consideration. The preliminary response of vendors suggests support for new and emerging avenues to distribute information respecting their products. Many vendors already maintain web sites and are looking to promote their use. Specific ideas to identify an editor‐in‐chief and an editorial board, establish and build the journal as a stand‐alone business, target participation from members of the larger medical physics community, develop a user profile for marketing purposes, and promote collateral growth within the ACMP are discussed. Some technical aspects respecting how the journal might operate are outlined. It is our hope to have a working demonstration of this journal and its proposed operation at the 1998 ACMP Board meeting.

## II. SURVEY OF CURRENT SOURCES OF CLINICAL AND SCIENTIFIC INFORMATION

Published by the AAPM, *Medical Physics* enjoys an international reputation for excellence among scientific journals. However, many valuable clinical papers which do not contain new science are rejected for review under existing editorial policies. The same can be said for *Physics in Medicine and Biology*, published in the UK by the Institute of Physics and Engineering in Medicine. Published by ASTRO, *The International Journal of Radiation Oncology Biology Physics* also enjoys worldwide respect. Physics articles are, however, limited and only a small percentage of medical physicists are members of ASTRO and receive the Journal. *Medical Dosimetry* is published by the American Association of Medical Dosimetrists. This Journal does not enjoy the reputation of the three journals first mentioned. In addition, few medical physicists are members of the AAMD and consequently receive the Journal. Proceedings published after clinical workshops are often of high quality; however, distribution is usually limited to participants in the conference. Task group reports address some clinical issues, but the scope of these reports is limited to the executive charge to the group. The investigation, drafting, and review process for these reports often takes years to complete. Proceedings, workshop reports, manuscripts rejected due to lack of space, and reports of individual projects practical in nature constitute a class of clinical information within the medical physics community which cannot find a timely, peer reviewed publication outlet which also reaches the general medical physics population.

## III. IDENTIFICATION OF THE TARGET AUDIENCE FOR THE JOURNAL

The potential target audience is composed of all practicing clinical medical physicists, and a limited number of radiation oncologists, radiologists, dosimetrists, and administrators. Currently, we estimate approximately 70% of the target audience has access to the Web, with the vast majority of these having no better than 28.8 K modem communications. While the entire ACMP membership is part of the target audience, ACMP members comprise only 10% or less of the target audience.

## IV. ALTERNATIVES TO AN INDEPENDENT JOURNAL

Suggestions include: 1) expand the ACMP, AAPM or IOMP newsletter to include peer‐reviewed clinical articles; 2) co‐publish clinical articles with *Medical Physics* or *Medical Dosimetry*; 3) co‐publish articles with *Administrative Radiology* or *Reports on Practical Oncology*. Each of these avenues were found to have significant drawbacks respecting cost, vendor interest, current publication intent, scope of distribution, and potential of cooperation with other organizations. In particular, it was expressed the AAPM would not be comfortable with incorporating this type of activity into *Medical Physics*. Also, none of the above offer the advantages of an electronic journal discussed below.

## V. SCOPE, COMPOSITION AND VENDOR SUPPORT OF A CLINICAL MEDICAL PHYSICS JOURNAL

Following is a suggested, but not exhaustive, list of the types of articles which might be published: 1) articles which describe a unique solution to a clinical problem for a specific patient, type of patient target volume, or piece of equipment; 2) equipment evaluation and review articles describing commissioning tests and techniques for clinical implementation; 3) articles which describe multiple types of equipment, often from different vendors, which must work together and communicate seamlessly; 4) health physics papers describing unique shielding or radiation protection problems; 5) papers dealing with Quality Assurance, Continuing Quality Improvement, and Total Quality Management; 6) articles dealing with administrative problems including staffing, space, equipment evaluation and purchase, project analysis, and resource planning; 7) review articles dealing with how different institutions and vendors approach similar clinical problems; 8) articles describing ways to implement and verify recommendations from scientific bodies (e.g., task groups) while recognizing specific equipment or clinical limitations; 9) business management articles addressing the changing reimbursement and management landscape and how individual medical physicists and the medical physics community might be proactive; 10) reviews of emerging technology, changing regulatory standards, and practice accreditation; 11) debates respecting clinical issues; 12) medical‐legal issues as they impact the practice of medical physics. The editorial board of the journal will be responsible to define the list of article types to be included in the journal.

An editorial by Gail Adams in Vol.1 No. 1 of *Medical Physics* (Jan/Feb 1974) describes the purpose of that journal, but also suggests other legitimate professional interests: “…professional purpose(s)… deals with the individual personal interests of the professional person and might include legislative lobbying, wage and salary negotiations, and unionization. We have no taste for these (in a scientific journal)…”

The proposed clinical medical physics journal would review and publish such articles which have been rejected by *Medical Physics* since inception, but nevertheless are legitimate concerns of professional life. The articles published in clinical and professional proceedings associated with the ACMP meetings number between 20 and 30 per year. An initial proposed goal which we believe may be achieved is to publish 15 such papers per quarter, or 60 papers per year. It was agreed the journal should accept articles from anyone, at least initially.

It is proposed the journal be published in quarterly issues with papers being posted to the current issue as soon as they are available. The journal might consist of seven sections: 1) Editorials; 2) Invited Papers; 3) Letters to the Editor; 4) Peer‐reviewed Articles 5) Peer‐reviewed Articles — sponsored by a specific vendor (that is, a “Vendors Corner”); 6) Continuing Education Articles — perhaps associated with CAMPEP; and 7) Calendar of Meetings. Serious consideration was given to a suggestion that an employment notification service be included as part of the journal. This idea was rejected as potentially too controversial for this new venture. However, we recommend the ACMP Board revisit the need for the ACMP to operate and publish placement notices for its members. We propose that vendors be allowed to sponsor papers for inclusion in a designated section of the journal. Sponsorship buys a one‐page advertisement to be included at the end of the paper and printed out upon retrieval. Also, the individual will receive a link to transfer to the web site of the vendor to view the latest offerings. This feature will exist in perpetuity for this paper at an initial cost of $500.00 to the vendor. This cost might be adjusted on a sliding scale according to the length of the article. These features allow the vendor to publish articles presenting technical and clinical specifics of a product, and to present timely information specific to the range of products offered by the vendor. Also, vendors will be listed along a bar on the left side of the journal page and vendor ads will flash every 20–30 seconds at the top or bottom of the page. This benefit to the vendor can only be accomplished using an electronic medium.

Vendors are going to support the journal on the basis of the number of “hits” or different individuals visiting the site and seeing the flashing ads each month. A good number in the industry is $0.25 per pair of eyes per month, or $0.75 per pair of eyes per quarterly issue, or $3.00 per medical physicist per year. A reasonable goal is 1000 “hits” per month from medical physicists. A vendor may be persuaded on this basis to support the concept of a journal during start‐up at a rate of $2,000.00–$4,000.00 per year, provided we demonstrate this level of activity. To attract younger medical physicists to the journal, continuing education features would be a prominent focus of the journal's mission.

## VI. ADVANTAGES OF AN ELECTRONIC JOURNAL AND TECHNICAL SPECIFICS

The advantages of an electronic journal include:1) lower operating and distribution costs; 2) worldwide access to the medium; 3) searching capabilities; 4) potential for multimedia capabilities including sound and moving pictures; 5) for those who do not subscribe, the potential for charging for articles on a flat‐fee per download basis; 6) there are some specific services which may be offered to supporting vendors which are only available if the format is electronic.

Articles will be submitted in Word‐6 or WordPerfect format with imbedded Excel, JPG or GIF files for tables and figures, receptively. They will pass through the entire review process in this manner. Once accepted for publication, the articles will be written by Adobe Acrobat into PDF format. They may be viewed online and in color, as they will appear on paper using this format. Adobe Acrobat Viewer is free software and is being distributed with the AAPM CD‐ROM to all AAPM members this month. It operates as a plug‐in for both the Netscape and Microsoft Web browsers. The articles may be downloaded and printed out by anyone who has access to the Web.

## VII. JOURNAL OPERATION SPECIFICS

The journal could operate in association with the ACMP Web page, which currently exists on the ACR server as part of the ACMP contract. Routine backup procedures and password protection are already in place for this server.

Journal operation will be under the director of an editor‐in‐chief and a managing editor. The editor‐in‐chief should be an ACMP member who enjoys universal respect within the medical physics community. Ideally, he/she should be a past chair of the ACMP, a past president of the AAPM, winner of national and international honors such as Williams and/or Coolidge awards, and demonstrate a long history of editorial involvement with *Medical Physics*, *PMB*, and / or *IJROBP*. The level of support for this position should be equivalent to that offered to the program of the current editor of *Medical Physics*, or $35,000.00 per year. This figure is based on six hours per week support for the editor and four hours per week support for a secretary. The editor‐in‐chief will be responsible for recruiting associate editors and peer reviewers, and determining the content of each issue. He/she will be responsible for assigning a retention period associated with the issues and articles before archiving to CD‐ROM or equivalent. The Managing Editor will be responsible for the operation of the journal and establishing vendor support. The Webmaster will be responsible for Web administration of the journal, including detailed standards for submission of articles. The financial matters for the journal would be handled by the Journal Finance Committee appointed by the ACMP Chairman during the three‐year start‐up period. These positions will be unpaid during the three‐year start‐up period. Total initial budget for this venture is $35,000.00 for support of the Editor‐in‐Chief, $5,000.00 for publicity, $5,000.00 for an assistant to the Webmaster, and $10,000.00 contingency fund to be used at the discretion of the Editor‐in‐Chief; total $55,000.00.

## VIII. JOURNAL ACCESS SPECIFICS

Journal access may be password protected, but the passwords could be offered for free during the start‐up period. Individuals may obtain a password online by answering a short questionnaire about their education and employment background. This information will be used to identify a user profile so vendors may be persuaded to invest money in the concept. After the three‐year start‐up period is complete, total access for download will be restricted to ACMP members or subscribers only, utilizing assigned password access. Subscription cost should be similar to the cost of ACMP membership for one year; current subscription cost for *Medical Physics* is $175.00 per year for individuals and $550.00 per year for libraries. However, any nonsubscriber may obtain a limited password by answering the questionnaire above; this individual will still be able to: 1) view, search and download the first three years of the journal; 2) view and search all abstracts for all years; and 3) download a specific paper for a user fee paid by credit card for a suggested price of $20.00. In comparison, to order a reprint from *Medical Physics* online costs $12.95 for shipping and handling and $15.00 royalty fee; total $27.95.

## IX. MARKETING THE JOURNAL

The free password to the journal marketing should be limited to all medical physicists within the United States. Notice of the existence of the journal may be sent online to medical physicists with email addresses. A one‐page mailing should be prepared and placed in the ACMP and AAPM mailings for fall of this year. The mailings should be an announcement of the existence of the journal and an invitation to submit papers. The AAPM mailing will cost the ACMP $300.00 insertion fee plus the cost of producing and shipping 4,500 fliers to the AAPM headquarters. This mailing should be performed three times. The ACMP could purchase the membership list from AAPM and perform a separate mailing. A second and third mailing will probably be required to expose fully the journal to the medical physics community. Hard copies of the journal should be placed at the ACMP booth during meetings, along with a free download service for medical physicists.

## X. COLLATERAL BENEFITS TO THE ACMP

The key to success of any company, organization or institution is the management of information, which is the lifeblood of its existence. Individuals and professionals cannot compete and survive in a hostile environment unless they have ready access to the information which allows them to survive in such an environment. The challenge to the ACMP at this point in time is either to grow or die. To provide the medical physics community with essential, timely medical physics professional and clinical information may be our best chance to double in size within the next 3–5 years. The *American Clinical Medical Physics Journal* (ACMPJ) may be the most cost‐effective and realistic vehicle to accomplish this aim. Although the cost of the operation during the start‐up period is significant ($ 55,000.00 per year for three years), the cost of walking away from this opportunity may be even greater. We hope to provide evidence of significant vendor support for this concept at the ACMP Board Meeting, as well as a working sample journal page.

